# “Diving in the deep-end and swimming”: a mixed methods study using normalization process theory to evaluate a learning collaborative approach for the implementation of palliative care practices in hemodialysis centers

**DOI:** 10.1186/s12913-023-10360-7

**Published:** 2023-12-11

**Authors:** Laura M. Holdsworth, Margaret Stedman, Erika Saliba Gustafsson, Jialin Han, Steven M. Asch, Glenda Harbert, Karl A. Lorenz, Dale E. Lupu, Elizabeth Malcolm, Alvin H. Moss, Amanda Nicklas, Manjula Kurella Tamura

**Affiliations:** 1 Division of Primary Care and Population Health, Stanford University School of Medicine, 3180 Porter Drive, Palo Alto, CA 94304 USA; 2 Division of Nephrology, Stanford University School of Medicine, Palo Alto, CA USA; 3 Center for Innovation to Implementation, Palo Alto VA Health Care System, Palo Alto, CA USA; 4 School of Nursing, George Washington University, Washington, D.C USA; 5 Division of General Internal Medicine, Duke University School of Medicine, Durham, NC USA; 6 Center for Health Ethics and Law, West Virginia University Health Sciences Center, Morgantown, WV USA; 7 Geriatric Research and Education Clinical Center, Palo Alto VA Health Care System, Palo Alto, CA USA

**Keywords:** End-stage kidney disease, Palliative care, Collaborative, Mixed methods, Normalization process theory, Hemodialysis, Practice change

## Abstract

**Background:**

Normalization Process Theory (NPT) is an implementation theory that can be used to explain how and why implementation strategies work or not in particular circumstances. We used it to understand the mechanisms that lead to the adoption and routinization of palliative care within hemodialysis centers.

**Methods:**

We employed a longitudinal, mixed methods approach to comprehensively evaluate the implementation of palliative care practices among ten hemodialysis centers participating in an Institute for Healthcare Improvement Breakthrough- Series learning collaborative. Qualitative methods included longitudinal observations of collaborative activities, and interviews with implementers at the end of the study. We used an inductive and deductive approach to thematic analysis informed by NPT constructs (*coherence, cognitive participation, collective action, reflexive monitoring*) and implementation outcomes. The NoMAD survey, which measures NPT constructs, was completed by implementers at each hemodialysis center during early and late implementation.

**Results:**

The four mechanisms posited in NPT had a dynamic and layered relationship during the implementation process. Collaborative participants participated because they believed in the value and legitimacy of palliative care for patients receiving hemodialysis and thus had high levels of *cognitive participation* at the start. Didactic Learning Sessions were important for building practice *coherence*, and sense-making was solidified through testing new skills in practice and first-hand observation during coaching visits by an expert. *Collective action* was hampered by limited time among team members and practical issues such as arranging meetings with patients. *Reflexive monitoring* of the positive benefit to patient and family experiences was key in shifting mindsets from disease-centric towards a patient-centered model of care. NoMAD survey scores showed modest improvement over time, with *collective action* having the lowest scores*.*

**Conclusions:**

NPT was a useful framework for understanding the implementation of palliative care practices within hemodialysis centers. We found a nonlinear relationship among the mechanisms which is reflected in our model of implementation of palliative care practices through a learning collaborative. These findings suggest that the implementation of complex practices such as palliative care may be more successful through iterative learning and practice opportunities as the mechanisms for change are layered and mutually reinforcing.

**Trial registration:**

ClinicalTrials.gov, NCT04125537. Registered 14 October 2019 – Retrospectively registered.

**Supplementary Information:**

The online version contains supplementary material available at 10.1186/s12913-023-10360-7.

## Background

Implementation science aims to close the research to practice gap by identifying effective strategies for implementing and sustaining evidence based practices [1]. Many frameworks, models, and theories stemming from a wide range of disciplines exist and can be used by implementers and researchers as a lens to help understand implementation processes and outcomes [2]. Use of such tools help make sense of empirical findings and find meaning among the chaos [3]. Theories, in particular, can be used to understand how and why implementation strategies work or not in particular circumstances as they *explain* causal mechanisms of implementation, rather than simply describe implementation processes [2]. Mechanisms can be defined as processes within a system that bring about or prevent change [4, 5]. Normalization Process Theory (NPT) is an implementation theory that focuses specifically on “how and why things become, or don’t become, routine and normal components of everyday work” ([5], pp. 535). NPT posits four mechanisms for how new practices become routinized: *coherence* (i.e. sense-making)*, cognitive participation* (i.e. enrollment and engagement)*, collective action* (i.e. enacting)*,* and *reflexive monitoring* (i.e. appraisal) (see Table 1 for construct definitions). These mechanisms are theorized as consistent and stable across instances of enacting practices and it has been used to evaluate numerous healthcare interventions [6].

**Table 1:** Description of Normalization Process Theory (NPT) constructs, mechanisms and guiding questions to operationalize the theory (from May et al., 2009) with exemplar study questions

NPT construct	Description of the mechanism	Operationalizing question
Coherence	Work that defines and organizes a practice as a cognitive and behavioral ensemble; i.e. sense-making	What is the work? E.g., What does ‘palliative care’ mean to clinicians?
Cognitive participation	Work that defines and organizes the individuals implicated in a practice; i.e. enrollment and engagement	Who does the work? E.g., Who will be involved in having goals of care discussions?
Collective action	Work that defines and organizes the operationalizing of a practice; i.e. enacting	How does the work get done? E.g., How do clinical teams identify seriously ill patients?
Reflexive monitoring	Work that defines and organizes the everyday understanding of a practice; i.e. appraisal	How is the work understood? E.g., What do clinicians think about using a palliative care approach in their routine clinical care?

Within healthcare, the implementation of palliative care for patients receiving hemodialysis has been limited, making this area ripe for intervention and evaluation [7]. Palliative care is an approach to care that is focused on quality of life and symptom management, and is often appropriate for patients with advanced illness [8]. The barriers to palliative care in dialysis are well documented and include underdeveloped models of care for seriously ill patients, lack of access to specialist palliative care, and misaligned incentives through Centers for Medicare and Medicaid Services (CMS) reimbursement structures [7].

To address some of these barriers, the Pathways Project learning collaborative was designed as a strategy to facilitate the implementation of palliative care best practices in hemodialysis centers [9]. Learning collaboratives are a well-established strategy for changing practice with evidence of effectiveness [10]. The collaborative focused on implementation of three palliative care practices: (1) screening to identify seriously ill patients using the “surprise question” (“Would I be surprised if this patient died in the next 12 months?”) [11], (2) conducting goals of care conversations and advance care planning (ACP) for future care options, and (3) providing palliative hemodialysis, defined as a reduced hemodialysis schedule for patients with less than one year to live, and a systematic hemodialysis discontinuation process for appropriate patients. Among participants in the collaborative, screening for serious illness was widely adopted and sustained, and documentation of advance care planning increased [12]. However, the practice of offering palliative hemodialysis was not systematically adopted.

In this study, we explore the mechanisms that underpin these observations of implementation and effectiveness outcomes. Focusing on mechanisms will lead to creating generalizable knowledge about *how* the learning collaborative led (or did not lead) to the adoption and routinization of palliative care practices within hemodialysis centers and in what contexts it is likely to work for future spread [13, 14]. NPT has been used previously within the area of palliative care and therefore is well-suited for this purpose [15, 16].

## Methods

### Design and theoretical framework

We used a mixed methods design to evaluate if and how a learning collaborative can lead to adoption and sustained use of palliative care practices for patients with end-stage kidney disease in hemodialysis centers. We used NPT as the theoretical framework to conceptualize the mechanisms of change and to underpin the design, data collection tools, and analysis [5]. NPT was selected because it can “[provide] a conceptual framework to assist in the understanding and explaining the dynamic processes that are encountered during the implementation of complex interventions” ([17], pp.2). Table 1 describes the four generative mechanisms of NPT and questions used to operationalize them [5]. These mechanisms are conceived of as dynamic, interacting within a social context to promote or inhibit implementation, embedding, and integration of complex interventions.

### Setting and participants

Ten hemodialysis centers representing three dialysis organizations and affiliated academic and non-academic nephrology practices took part in the collaborative. Centers were located in the New York City, Denver, and Dallas metropolitan areas. Care in these centers was directed by nephrologists within a multidisciplinary team (e.g., nurse practitioner, nurse, social worker, dietitian), as is typical for hemodialysis centers. Multiple providers practiced within each center, though not all providers participated directly in the collaborative.

### Intervention

**Fig. 1:**
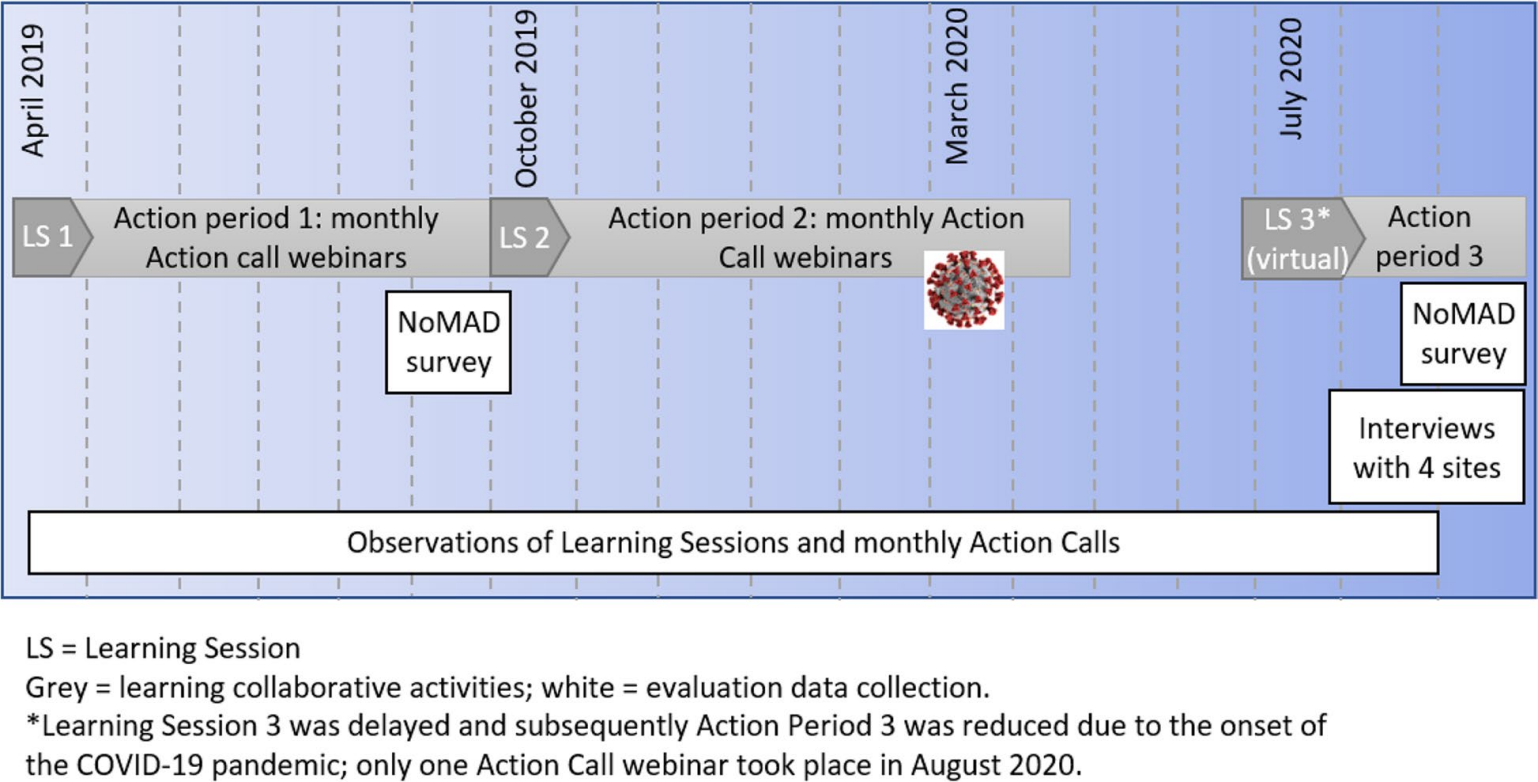
Timeline of collaborative activities and evaluation data collection

A detailed description of the intervention development and collaborative structure has been published [9]. Briefly, the Pathways Project identified a change package (see supplement) consisting of 14 evidence-based best practices in palliative care and prioritized three palliative care practices for implementation, described in the Background section. Together, the three core practices constitute a complex intervention, in that it consists of multiple interacting components that can each be customized to suit local needs, affects various provider behaviors, and may influence numerous outcomes [18]. The implementation strategy consisted of an Institute for Healthcare Improvement Breakthrough Series collaborative which ran from March 2019 through August 2020 [19]; Fig. 1 shows a timeline of learning collaborative activities and data collection. Implementation teams consisting of a nephrologist or advance practice provider and two to four additional multidisciplinary team members from each hemodialysis center participated in the collaborative. The Breakthrough Series is designed for healthcare organizations that want to improve certain practices and provides a structure for participants to learn from each other and from experts. The collaborative curriculum covered the change package best practices, serious illness communication skills, and quality improvement methods. The collaborative structure included three Learning Sessions, monthly Action Calls following Learning Sessions, regular support from a quality improvement expert, and coaching in goals of care conversations from a nephrologist dually boarded in palliative care (AHM). The first two Learning Sessions were conducted in person over two days in April and October 2019, and the third was conducted virtually in July 2020 over three days due to the COVID-19 pandemic. The monthly Action Calls checked in on site activities and provided feedback on site performance in a group format.

### Data collection

Learning Sessions and monthly Action Calls were observed by one researcher (LMH) to capture the content and structure of collaborative education and activities over time, as well as participation and engagement by implementers from hemodialysis centers. Short handwritten notes taken during observations were transcribed into narrative format immediately following observational sessions for analysis.

At the end of the implementation period, a sub sample of four hemodialysis centers were selected as cases for additional qualitative in-depth study. The centers were selected for diversity of organization, region, and implementing team personnel, and all implementers within each center were invited to participate in an interview [20]. The interview topic guide was informed by NPT and implementation concepts, and also included questions to probe deeper into observational data (see supplement). Questions were geared towards understanding the mechanisms of change, conceptualized as specific events, activities, or moments that led to changes or shifts in thinking or practice. Interviews were conducted by an experienced qualitative health services researcher who had observed collaborative activities and thus was known to most participants prior to the interview (LMH). A nephrologist (MKT), who did not previously know the participants, also joined for at least one interview per site to probe on clinical practice issues. Following each interview, key points were summarized and summaries reflecting individual perspectives and consensus among individuals within a center were sent to participants as a member checking exercise to confirm understanding and validate conclusions [21]. Interviews were recorded with permission and transcribed for analysis.

The Normalization Measure Development (NoMAD) survey, which assesses NPT constructs [22], was administered to implementing teams (approximately 3–5 members per site) at all ten centers during early implementation (September 2019), and at the end of the collaborative (September 2020). The NoMAD survey measures the relative importance of the NPT constructs and subconstructs in achieving sustained practice changes [22]. Because each practice is distinct and mechanisms, components, and investments may vary across them, following advice from the survey developers, we separately assessed NPT constructs for each practice. In order to reduce the overall length of the survey, we included only one question for each subconstruct in the survey (see supplement). Each item on the NoMAD uses a five-point Likert scale, with an additional option to indicate whether an item is not relevant. To obtain construct scores, we averaged Likert scale scores for NPT sub-constructs. Higher scores indicate that a new practice was more likely to be implemented and sustained. Participants were also asked to rate how much each of the best practices was part of their current practice.

### Analysis

Observation narratives and interview transcripts were uploaded into NVivo (released March 2020) for data management and analysis. Audio for all interviews was listened to prior to coding to check for accuracy and improve understanding. Thematic analysis used both a deductive and inductive approach. An initial codebook was created deductively using codes derived from NPT constructs and subconstructs, implementation outcomes [23], change package best practices, and topic guide questions. Inductive codes were added to the codebook during coding as they emerged. One researcher (LMH) coded all the data. As a check of our understanding of identifying NPT constructs, a second researcher with qualitative training and implementation science experience (ESG) coded five interviews. The two coders met regularly during coding to discuss understanding and meaning of NPT within the data. This process revealed that constructs often appeared together; i.e. people making sense of how practices are different (*differentiation*) was sometimes the product of working collectively as a team and discussing practices (*communal specification).* The remaining transcripts were re-coded by the lead researcher (LMH) to check for any missed constructs. Coded data was then organized into matrices to look for relationships between constructs, how they related to the adoption and sustainment of new practices, and patterns across sites. Thematic findings were tested by searching for negative evidence [24].

Frequencies and percentages were used to summarize staff characteristics among survey participants during early and late phases of implementation. For each of the three best practices we computed a construct score by averaging related items scores together. Construct scales were computed for each staff member of the site participating in the NoMAD survey. To compare the construct scale across phases of the study, we performed a generalized estimating equations (GEE) model of the construct scale (dependent variable) and phase of implementation (independent variable), adjusting for the correlations within site with robust standard errors. Surveys were anonymized so we were unable to adjust for correlation at the staff member level. We selected a log transformation to reduce the skewness in the data.

Findings from the qualitative and quantitative methods were triangulated at the interpretation stage by checking for convergence or dissonance in findings [25].

The study was approved by Stanford University Institutional Review Board, IRB 51404 and by the George Washington University (GWU) Institutional Review Board, IRB 180679.

## Results

Three Learning Sessions and 14 monthly 1-h Action Call webinars were observed for a total of 49.5 h between April 2019 and August 2020. Interviews with implementers were conducted via Zoom between August and September 2020 and the median length was 52 min (range 23 to 65 min). Professional groups interviewed included nephrologists (*n* = 4), social workers (*n* = 4), nurse practitioners (*n* = 2), and nurse/nurse managers (*n* = 4); at least one participant per site also occupied a leadership role, such as medical director or director of nursing. In addition, the Pathways quality improvement trainer was interviewed four times periodically during the collaborative to provide insight into hemodialysis centers’ progress over time. NoMAD surveys were completed by 30 individuals during early implementation and 29 during late implementation. Table 2 presents the characteristics of survey respondents.

**Table 2:** Characteristics of hemodialysis center staff who participated in the Pathways collaborative and completed NoMAD surveys in early and late implementation

Characteristics of staff	Early implementation *N* = 30 (%)	Late implementation *N* = 29 (%)
Provider type:		
Social Worker	13 (43.3%)	10 (34.5%)
Nurse	6 (20%)	7 (24.1%)
Nurse Practitioner/ Physician Assistant	5 (16.7%)	5 (17.2%)
Nephrologist	2 (6.7%)	5 (17.2%)
Other	4 (13.3%)	2 (6.9%)
Number of years’ experience^a^:		
< 1 year	3 (10.3%)	2 (6.9%)
1–2 years	6 (20.7%)	4 (13.8%)
3–5 years	10 (34.5%)	6 (20.7%)
6–10 years	3 (10.3%)	9 (31.0%)
11–15 years	3 (10.3%)	5 (17.2%)
> 15 years	4 (13.8%)	3 (10.3%)
Organization^b^		
A	10 (33.3%)	12 (41.4%)
B	11 (36.7%)	8 (27.6%)
C	9 (30%)	9 (31%)

^a^1 missing response for early implementation, *N* = 29

^b^Organizations have been deidentified

**Fig. 2:**
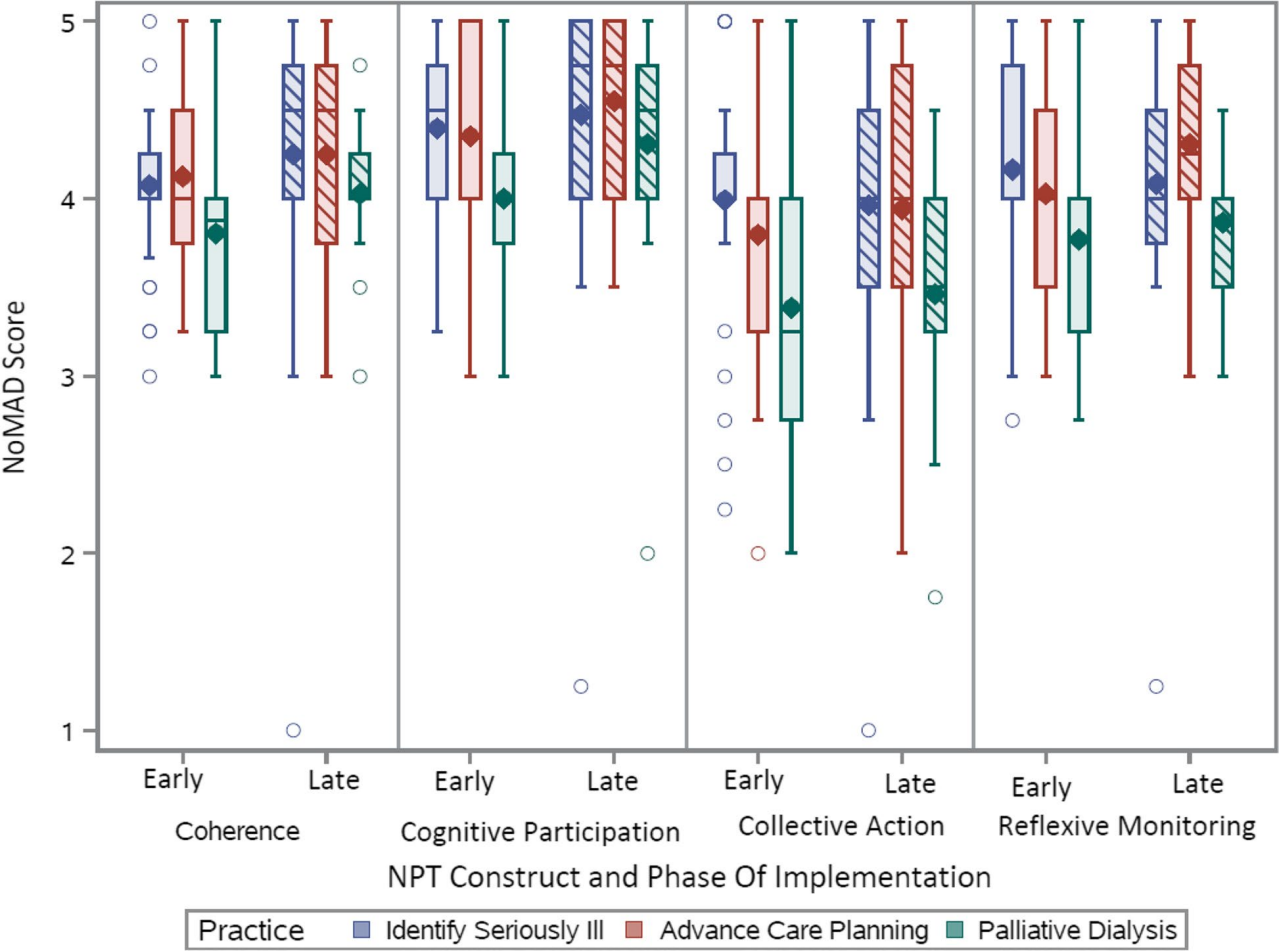
Boxplot of NoMAD scores for each of the three palliative care practices by NPT construct for early and late implementation. The box indicates the interquartile range. The whiskers represent 1.5 times the interquartile range or the boundary for identifying outliers. The symbols indicate the mean and o indicates outlying values

Figure 2 shows that construct scores for each of the three best practices were moderate or high (i.e. scores above 3) at the early implementation phase. Scores were highest for *cognitive participation,* and lowest for *collective action*. Overall, scores for each mechanism were either largely stable or trended towards a modest increase for each practice by late implementation. Palliative hemodialysis had the lowest overall scores at early implementation. Scores for this practice increased at late implementation, but remained lower compared to the two other best practices. Individual scores for subconstructs for each practice are presented in supplemental material. Table 3 indicates that identifying seriously ill patients and having goals of care conversations were more commonly considered a part of current practice after five months of participating in the collaborative (i.e., early implementation), whereas palliative hemodialysis had more limited uptake, even at the end of the collaborative. This perception of routinization of practices for the first two practices is reflected in the relatively high NoMAD scores in Fig. 2.

**Table 3:** Survey responses of hemodialysis staff to questions about how normalized palliative care practices are in current work

Palliative care practice	Early implementation *N* = 30 Mean (SD)	Late implementation *N* = 29 Mean (SD)	*p*-value
Do you feel identifying seriously ill patients is currently a normal part of your work?	7.7 (2.0)	8.5 (1.5)	0.17
Do you feel advance care planning is currently a normal part of your work?	7.8 (2.3)	8.4 (1.5)	0.29
Do you feel palliative dialysis is currently a normal part of your work?	3.7 (3.3)	6.0 (3.3)	0.01

Survey response range: 0 = not at all a part of work to 10 = completely a part of work

*P*-values based on the Wald Chi-Square test from a GEE model comparing the difference between early and late implementation scores. We adjusted for the correlation of the repeated measures within site

Using NPT, we explored how teams learned, implemented, and embedded palliative care practices. We first present the findings organized under NPT constructs and then propose a model for explaining how the collaborative led to observed changes. Though results are organized by NPT construct, it should be noted that, as indicated by our coding process, mechanisms are often layered as people integrate new practices.

### Coherence – making sense of palliative care through practice

The first Learning Session introduced participants to the surprise question to screen patients for serious illness, and serious illness communication skills through role playing. Learning these skills were the first bricks on which participants began to build a scaffold of new practice:

*it feels so much more intuitive now, that I don't necessarily think about, "Oh, I learned this in training." But, I think a lot of the role play exercises at the beginning, […] I think those were incredibly helpful. (Interview 8, social worker)*

A pivotal point in sense-making for many participants came during a coaching site visit from the Pathways nephrologist. During these visits, the nephrologist would conduct a goals of care conversation with seriously ill patients from the center while the center’s implementation team would observe. Participants described techniques they picked up that they then adopted for their own practice, such as asking questions when patients offered resistance, or taking the *“mystery out of [the conversation]” (Interview 4, nephrologist*) by giving patients the conversation guide that the nephrologist used.

*I think it was really helpful having [the nephrologist] come here and talk to our side with a few of our patients. [...] Just hearing the language that he used, that it wasn't sugarcoated really. I mean, he was very honest with the patients about what it meant, but still very kind. (Interview 9, social worker)*

These opportunities for observation and coaching that took place over the course of the collaborative became a key source for understanding the practical application of Learning Session information and a crystallizing point for many as they differentiated between their own attempts at conversations and how resistance from patients could be overcome.

Likewise, attempting a goals of care conversation was fundamental to developing participants’ communication skills as it did not always make sense until they tried it:

*It's very interesting that [the social worker] said, "Oh, [the physician has] talked to all these patients [about goals of care]," after it dragging on for months and not going. So I think it's just having people jump into the deep end a little bit and start having these conversations, that it gets easier. (Interview 2, nephrologist)*

### Cognitive Participation – valuing palliative care

Participation in the collaborative, at both the hemodialysis center and individual level, was voluntary and thus all participants were motivated to improve their serious illness communication skills. This motivation was evident in interviews and is reflected in the high NoMAD scores for cognitive participation across the three practices in the early implementation period. Several participants referenced personal experiences with seriously ill family or friends as sources of this motivation:

*I had a personal experience with my dad, and having gone through that traumatic ICU experience and not knowing his wishes and having to speak for him. […] So I went through that whole process of being involved with that end of life and just how important it was for myself and my family and having that support. So I guess that personal experience led me to have a deeper appreciation for hospice care or end of life care (Interview 1, social worker)*

While prior personal or professional experiences of serious illness care were motivating for many of the early implementers, seeing local process data collected over the course of the collaborative that was prepared by the Pathways team and fed back to sites also helped to get buy-in from other in-center nephrologists in the latter stages of the project:

*[The social worker] talked about having presented about the project the previous day to [the medical director] and he was really happy with the results and is now totally “bought in to it”, whereas at the start he wasn’t. (Observation, LS3, day 3)*

During Learning Sessions, implementing teams, which were typically led by a nephrologist, worked together to develop a team understanding of the new work, to develop plans for how they would routinely identify seriously ill patients using the surprise question, and who would be involved in having goals of care discussions with patients.

### Collective action – working together to deliver palliative care best practices

Following Learning Session 1, participants returned home to test out their new skills to mixed success. Most participants quickly enacted the processes they had planned during Learning Session 1 to use the surprise question on a monthly basis for identifying seriously ill patients. Typically, the surprise question was used prior to rounds or during multi-disciplinary care plan meetings. In contrast, teams had limited success with initial attempts to arrange goals of care conversations with patients. Approaches to having conversations varied by center: one center adopted the chairside approach as they had no conference rooms for family meetings, whereas others found that patients seemed uncomfortable at the chairside and therefore preferred scheduling private meetings.

A frequent barrier to engaging in goals of care conversations was the availability of nephrologists. For nephrologists participating in the project, the major limiting factor was finding time to schedule appointments. For nephrologists who were not part of the implementation team, but who cared for patients in the participating hemodialysis center, there were various other issues. Implementing teams reported that some nephrologists were supportive of the project and identified their seriously ill patients, but a few were noted by their peers to have beliefs incongruous with the project. Implementing teams tried to spread practices to willing providers; for those unwilling, they “*agreed to disagree*” (Interview 6, nurse practitioner). This was a barrier because social workers and nurses, who typically covered the patients of multiple nephrologists- including those not directly involved in the project, perceived that they needed permission from nephrologists to have discussions about serious illness with their patients.

The healthcare system structure was noted to be a barrier in the adoption of palliative care practices directly and indirectly. Specifically, the change package encompassed processes to de-escalate care, which implementers noted was at odds with the acute care centric health care system; therefore, the implementation of the palliative care practices were seen to be entirely driven by provider motivation. This reliance on provider recognition of deterioration and subsequent activation was articulated clearly by one nephrologist who contrasted palliative care discussions to conversations about starting dialysis with patients approaching kidney failure in which patients can’t start dialysis without a preceding conversation. The same hard stop was perceived as non-existent for goals of care discussions and thus such conversations were only initiated if a nephrologist opted to do so:

*We're handcuffed clinically until a conversation [about starting dialysis] is had. That's almost never true of palliative decision-making. You can always just keep going forward on the path that you're going. There's never a moment where like, "All right, we can't [go forward until we talk about it.] " There's a lot of moments where it makes sense to [have goals of care discussions], but you never have to do it. (Interview 11, nephrologist)*

With regards to palliative hemodialysis, payment models were perceived to disincentivize reduced hemodialysis schedules even when desired by patients. Though individual nephrologists were supportive of offering palliative hemodialysis to appropriate patients, this was usually only offered ad hoc as it was perceived that doing so systematically would be viewed negatively by partner nephrologists and hemodialysis center management:

*We've got a couple of patients that are unofficially dialyzed in a quasi-palliative way that are getting less dialysis by the book than other patients. But that is not something that we've standardized and worked out within the practice. And I feel a little uncomfortable about that because it looks like I've just gone rogue and done my own thing. (Interview 4, nephrologist)*

Despite widescale acceptability among implementers of the value of palliative care practices, participants expressed that this was necessary but not sufficient, and that systems still needed to be created so that tasks, such as generating and reviewing a list of patients, were “*hardwired*” (*Interview 2, nephrologist*) into people’s roles. Several sites acknowledged that sustaining the changes would require expanding the practices beyond the initial implementation team as one site noted that implementation team members were starting to feel burnt out:

*As we got towards the end of this, the questions and the conversations that we started to have were “this is a lot. We're tired. We're the only ones pushing this. How do we get some support here?” […] That's what I got from the staff is that they wanted us to continue, but they didn't want to be in it alone anymore. (Interview 6, nurse practitioner)*

### Reflexive monitoring – the crystallization of palliative care practices

There were various points in the journey of learning and enacting new skills where a new, patient-centric culture of practice seemed to crystallize based on individual and collective appraisal. Perceiving a positive impact on patients was particularly meaningful for social workers and nurses, and implementers were often able to identify at least one patient who had a positive outcome which implementers felt good about. Participants who were able to apply their new skills in unexpected or spontaneous situations felt particularly reassured and shared their positive experiences with others:

*When I did [the first] assessment [on a patient], […] the conversation about his wishes and hospice and palliative care now that- he was extremely sick, I think he may have lived three treatments- but that conversation just flowed naturally. I even came back and told the social worker, "Holy cow, I can't believe that." It just happened and that was cool. You didn't have to think about it. (Interview 3, nurse manager)*

This ‘feel good factor’ helped reinforce new practices and also helped people to identify that the quality of their practice was what mattered and had improved.

For others, particularly physicians, seeing data on the effectiveness of their work was reinforcing, particularly data that showed the high level of accuracy of the surprise question for identifying patients likely to die*.* For a few participants, the small tests of change approach to improvement which they learned during the collaborative was helpful for identifying improved practice, but largely did not appear to be widely utilized. Instead, process data collected by the Pathways team, analyzed, and shared with participants seemed to have higher value for encouraging change.

### Collaborative model of change using NPT

**Fig. 3:**
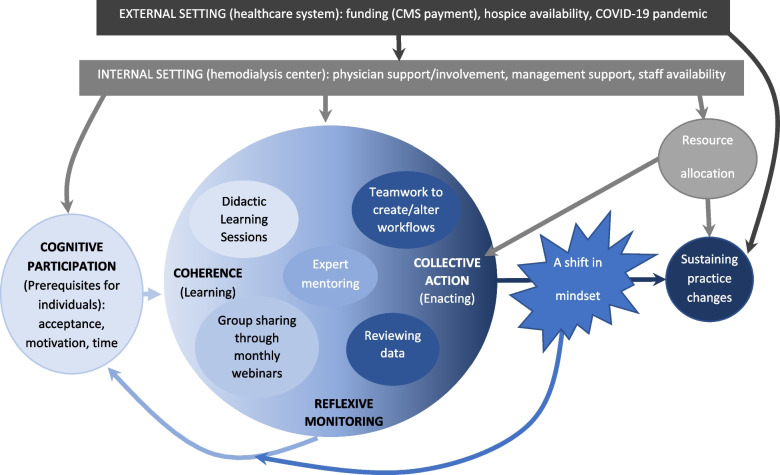
Model of change for implementing palliative care practices in hemodialysis centers through a learning collaborative

Figure 3 illustrates a model for change using NPT and implementation science concepts (external and internal setting from the Consolidated Framework of Implementation Research (CFIR) [26]). *Coherence, cognitive participation, collective action*, and *reflexive monitoring* were all interconnected mechanisms that were layered and mutually reinforcing throughout the implementation process. Participants appeared to have a high level of *cognitive participation* prior to the first Learning Sessions, which was reinforced by seeing the impact of new skills on clinical care and improvement in collaborative metrics. Though subconstructs related to *coherence* were an essential first step towards learning a new practice, that learning did not solidify until people had the chance to enact and reflect on those practices, which in turn changed their thinking and understanding of serious illness communication and care (i.e. mindset), thus leading to sustainable changes in communication practice. This shift towards a patient-centric mindset focused on prioritizing patient goals was described by participants in different ways, such as recognizing that goals of care conversations are an ongoing process and not a one-off, accepting that patients might want aggressive care even if physicians perceived it as medically nonbeneficial, and picking up and acting on cues that patients were concerned about their health. This shift, and the layered way in which it was brought about, was articulated by one social worker who described how her team’s understanding of quality of care for seriously ill patients had shifted because of the collaborative process, and the subsequent impact that this had on how the team worked together to deliver care:

*I feel like, now, I'm more comfortable discussing patient concerns with the doctor, because I know that, sometimes, […] the doctor can be so concerned about preservation of life, both for the statistical reasons for the clinic, but also because that's what they're trained to want for the patient. But now, I feel like I have the tools to say, "Well, it's not all about preserving life and extending life. Sometimes it's about quality of life." I feel like I now have the terms, and capacity to have that conversation with the doctor, in a way that I'm more confident going into it. […] I definitely think that it has helped that [the nephrologist] had the training [as well]. Because, I think he can hear me, not physically, but metaphorically, he can hear me when I say those kinds of things. Whereas, I feel like some of the other doctors [who weren’t trained] don't hear it, because it's just so foreign to them. (Interview 8, social worker)*

However, a shift in mindset was not sufficient to integrate new practices; tasks also had to be distributed among the multidisciplinary team. Task distribution was dependent on material, human, and time resources. In particular, the lack of adoption of palliative hemodialysis demonstrates that it is not possible to adopt a new practice if features of the external setting (i.e. the CMS reimbursement structure) are not conducive. The final steps to sustained practice change required a shift in mindset and resource allocation within a conducive wider environment; this pattern was evident across all three practices which ranged from successful (i.e. identifying seriously ill patients) to failed implementation (i.e. palliative dialysis).

## Discussion

Using NPT, we set out to assess how a learning collaborative as an implementation strategy led to the adoption and routinization of palliative care practices within hemodialysis centers. A strength of this study was the longitudinal collection of data that enabled us to develop a picture of implementation over time. Few studies have examined implementation using NPT longitudinally over the lifespan of an intervention [6, 27]. In doing so, we were able to see the evolution of implementer’s attitudes and understanding of the practices from the beginning to the end, helping to identify a clear shift in mindset about the practice of palliative care and the mechanisms that led to that shift. This shift in mindset led implementers to rethink the palliative care practices they had learned during the collaborative and reframe how they could be made into workable team practices. Additionally, our model of change was developed from observing the implementation of three different supportive care practices, which each achieved different implementation outcomes and thus the model represents processes that are common to both successful and failed implementation.

The mechanisms posited in NPT were evident in the process of implementing palliative care practices and were non-linear, as has been identified elsewhere [28, 29]. Collaborative participants were potentially unique among hemodialysis center providers in that their *cognitive participation* was high at the start as evidenced in both interviews and NoMAD scores; they participated because they believed in the value and legitimacy of palliative care for patients receiving hemodialysis. Indeed, participants may even be considered “early adopters” of palliative care practices in hemodialysis settings [30]. The Learning Sessions and monthly Action Calls were only part of the process of understanding new practices (*coherence*); sense-making was solidified through testing new skills in practice and first-hand observation during coaching visits by an expert. While *collective action* was initiated during the Learning Sessions and planning for who will do the work, task negotiation and division of work between team members happened while trying to implement and test new skills. Challenges in the feasibility of having goals of care conversations related to time, scheduling, and clinic space were commonly reported, which aligns with previous research, and is likely reflected in lower *collective action* NoMAD scores [31]. *Reflexive monitoring* in the form of identifying positive benefit to patient and family experiences was a key source of shifting minds from a disease-centric model of care towards a patient-centered model that prioritizes patient goals irrespective of disease-based metrics. A practical recommendation for implementers is to create repeated opportunities for teams to learn, enact, and reflect on new practices as it is the iterative nature of the collaborative process that helped people to understand and integrate new practices.

Regarding the NoMAD survey scores, there were relatively high scores with minimal change over time for the two practices of identifying seriously ill patients and having goals of care discussions, suggesting that participants felt that these two practices were stable from early to late implementation. However, while clinicians thought that their work in these two areas did not necessarily change, the quality of their work as reported in interviews improved. Our analysis of mechanisms indicates that the collaborative may have been an effective implementation strategy for creating this shift. Previous research using NPT conducted in an acute setting found that clinicians perceived that end of life care was at odds with their recovery focused mental model of care and thus found it difficult to make sense of it [16]. Hemodialysis centers may be similarly recovery focused as most patients receiving dialysis die without receiving palliative care or hospice despite having significant need for such services [32, 33]. Our findings suggest that the collaborative model, with an iterative learning process, supports the mechanisms needed to create a new mindset for some clinicians which is necessary for the sustained implementation of palliative and end of life practices in a recovery-focused environment.

Despite improvements made in identifying seriously ill patients and discussing goals of care as identified in our effectiveness study [12], there were systematic barriers to palliative hemodialysis and access to hospice care. Our data indicate a clear influence of the internal and external settings on the work that people do; this was particularly evident for palliative hemodialysis which was noted to be misaligned with CMS reimbursement for dialysis and therefore deprioritized among hemodialysis center leaders. This perception is likely reflected in the NoMAD scores on collective action which showed minimal change from early to late implementation. Widespread adoption of palliative dialysis is not likely to happen until this practice is recognized as patient-centered treatment by professional organizations and CMS, suggesting that implementation strategies needed for this practice change relate to financial strategies (i.e. reimbursement models) or changing health system infrastructure [34, 35]. Alignment of incentives within healthcare has been identified as crucial for leadership support: “alignment with national priorities, quality strategies, financial incentive systems or performance management targets may mobilise leadership to promote facility engagement in [quality improvement collaborative] programmes” ([36], pp.12). Our data suggest that the mechanism of *collective action* was influenced by external factors in ways that competed with learning collaborative efforts. Despite this, context is not well conceptualized in NPT, and has similarly been critiqued as problematic [36, 37]. Efforts to extend NPT have attempted to account for the real-world context in which work takes place and is an essential improvement to NPT, as is the development of an NPT codebook to help researchers conceptualize context as part of the analytic process [38–40].

### Limitations

Because of the COVID-19 pandemic, we conducted interviews via video instead of in-person site visits. In person visits might have offered additional insights into how practices were implemented. In terms of using NPT as a codebook, while the constructs and subconstructs are seemingly distinct, we identified significant overlap when operationalizing them, which has also been reported elsewhere [15, 27]. For example, ‘prior personal experiences’ both served to legitimize palliative care practices (*cognitive participation*) and helped people to make sense of them (*coherence*). Analysis was completed prior to the publication of the NPT codebook [40]. While our codebooks aligned seamlessly on the mechanism domain (we coded at both the construct and subconstruct level where possible), our groupings of discrete codes for context and outcome were different as we utilized constructs of inner and outer setting from the CFIR and Proctor et al.’s framework of implementation outcomes [23, 26]. While many of the same concepts are represented in both codebooks, the final organization of context and outcomes in the model may have been worded differently had the NPT codebook been used. Two sites at early implementation and three sites at late implementation returned only one NoMAD survey and therefore may provide limited insight into the implementation process of the center as a whole. In order to reduce the overall length of the NoMAD survey, we dropped one question for each of the four subconstructs that have two dimensions/questions (relational integration, skill set workability, contextual integration, reconfiguration). We may have therefore missed dimensions of those subconstructs that may have affected overall construct scores; summing subconstructs to arrive at construct scores aimed to minimize the loss of this data. We were not able to assess how many implementers were eligible for the survey at each site; the number of implementers eligible also changed at each time point due to staff turnover. The relatively small change in NoMAD scores over time suggests it may not be a sensitive tool for assessing change over time among a small sample of early adopters; additional studies in other contexts is needed.

## Conclusions

NPT was a useful framework for understanding how and why complex new practices become adopted and embedded, or not. We found a dynamic, layered relationship among the mechanisms which is reflected in our model of implementation of palliative care practices through a learning collaborative. *Cognitive participation* was typically a precursor to learning, and *coherence* was solidified through *collective action* and *reflexive monitoring* which in turn enhanced *cognitive participation.* The mechanism of *collective action* was influenced by factors outside of the learning collaborative, such as CMS policy and access to specialist palliative and hospice care, whereas the other mechanisms did not appear as clearly linked to external influence. These findings suggest that the implementation of complex practices such as palliative care may be more successful through iterative learning and practice, such as in a collaborative approach, as the mechanisms of behavior change are layered and mutually reinforcing.

### Supplementary Information

**Additional file 1.**

## Data Availability

The datasets used during the current study are available from the corresponding author on reasonable request.

## Abbreviations

ACP: Advance care planning
CMS: Centers for Medicare and Medicaid Services
NPT: Normalization Process Theory
