# Supplementary material for: “Diving in the deep-end and swimming”: a mixed methods study using normalization process theory to evaluate a learning collaborative approach for the implementation of palliative care practices in hemodialysis centers
Source: BMC Health Serv Res. 2023 Dec 11;23:1384. doi: 10.1186/s12913-023-10360-7 (PMC10712060; doi:10.1186/s12913-023-10360-7)
Supplement: Supplementary file 1 — Additional file 1. [file 12913_2023_10360_MOESM1_ESM.docx]

Pathways Project Change Package


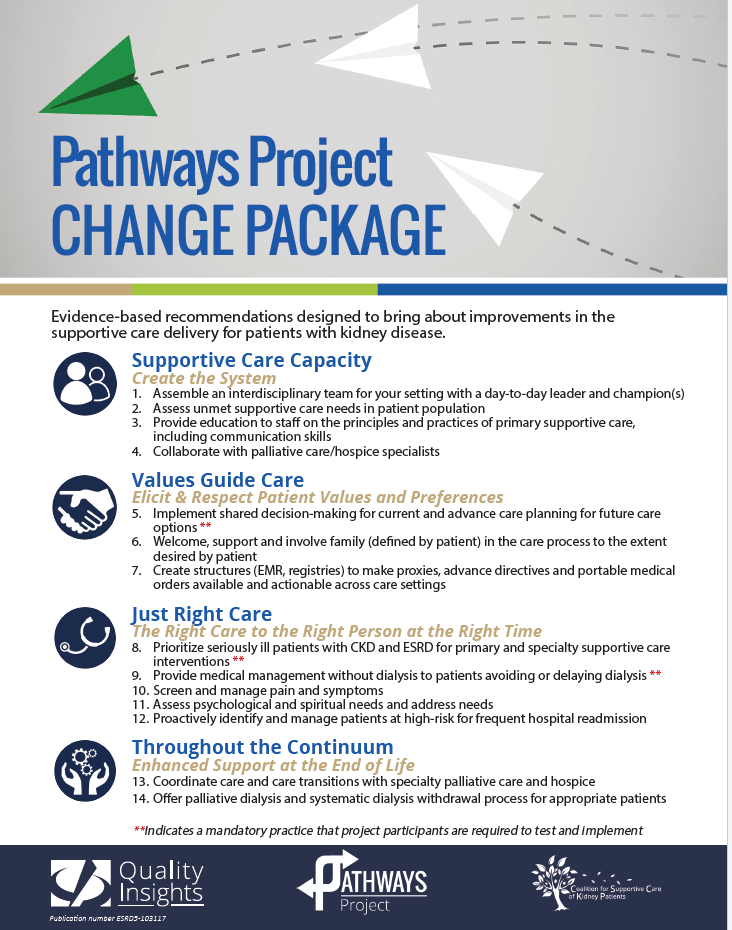


Topic guide for interviews with implementers

| Main question | Prompts/Probes |
| --- | --- |
| **Background: I’d like to start with a little bit of background information about yourself.** | |
| First, could you please tell me a bit about yourself, such as your role in dialysis center, years’ experience, how long you’ve been at this dialysis center. | Have you had any formal palliative care training?  What you enjoy most about your job? |
| For site lead ONLY: Can you tell me more about your center (e.g. staffing, organization type) | How was it decided who should participate in the project? |
| **Pathways collaborative structure: Thanks for that information. I’d now like to talk briefly about the structure of the Pathways project collaborative.** | |
| Did you participate in the:   - Learning Sessions? - Monthly webinar Action Calls? - Have a site visit from the Pathways team? | What did you think about them?  Did you find them helpful? |
| Overall, what did you think about the format of the collaborative? | What was the most useful part of the collaborative?  What did you think about the small tests of change (i.e. learning about QI, PDSA process)?  Did you learn from other sites?  Were the data reports from the Pathways team helpful? In what ways? How did you use them? |
| **Change package: Now I’d like to talk about what changes you’ve made during the Pathways project and in what ways the project helped to bring about those changes.** | |
| Starting with identifying seriously ill patients, how have you been able to do that in your center? | What changes did you have to make to accommodate this new practice?  Is it a new process or part of existing process (i.e. admission, regular review)?  What barriers to implementation did you encounter?  What helped to implement those changes? |
| Thinking about goals of care conversations, how have you been able to do that in your center? | What changes did you have to make to accommodate this new practice?  Is it a new process or part of existing process (i.e. admission, regular review)?  What barriers to implementation did you encounter?  What helped to implement those changes?  How do you feel now about having serious illness/ goals of care conversations?  How often do you have these types of conversations? |
| What other elements of the change package have you implemented, if any? | Why were those elements chosen?  How did those elements fit with your work?  Have you implemented palliative dialysis? If so, how? Or why not?  Have any new connections with hospice or palliative care providers been established? |
| Can you think of any point where you had an ‘aha’ moment, where something you learned crystallized for you? | What was it? Why was it so meaningful to you? |
| Overall, how successful do you think you’ve been in implementing the change package? | What contributed/ hindered that success?  Do you think you’re going to be able to sustain the changes that have been made? Why/ why not?  How much did the COVID epidemic impact your success? |
| **Lessons learned: Lastly, I’d like to talk about lessons learned.** | |
| What do you think dialysis centers need to know or consider if they were to implement the change package? | How does the current CMS payment model impact on this work?  Is there anything CMS could do to support serious illness/ goals of care conversations? |
| What would you suggest changing about the Pathways project? |  |
| Is there something else you’d like to tell me about that I haven’t asked about? |  |

NOMAD Survey modifications

Original survey: Finch, T.L., Girling, M., May, C.R., Mair, F.S., Murray, E., Treweek, S., Steen, I.N., McColl, E.M., Dickinson, C., Rapley, T. (2015). NoMAD: Implementation measure based on Normalization Process Theory. [Measurement instrument]. Retrieved from <http://www.normalizationprocess.org>.

The below survey is representative of how the NoMAD survey was customized for each of the three palliative care practices. The grey highlighting shows which questions were omitted from our version to reduce overall length. The original NoMAD survey included two questions for each of the following subconstructs and we opted to include only one question for each subconstruct: relational integration, skill set workability, contextual integration, reconfiguration.

| Please think about your practice of [palliative care best practice] for this section. If you have **not yet implemented** this component, check the box and skip this section. [box] | Strongly agree | Agree | Neither agree nor disagree | Disagree | Strongly disagree |  | Not relevant |
| --- | --- | --- | --- | --- | --- | --- | --- |
| I can see how [palliative care best practice] differs from usual ways of working. |  |  |  |  |  |  |  |
| Staff in this organization have a shared understanding of [palliative care best practice]. |  |  |  |  |  |  |  |
| I understand how [palliative care best practice] affects the nature of my own work. |  |  |  |  |  |  |  |
| I can see the potential value of [palliative care best practice] for my work. |  |  |  |  |  |  |  |
| There are key people who drive [palliative care best practice] forward and get others involved. |  |  |  |  |  |  |  |
| I believe that participating in [palliative care best practice] is a legitimate part of my role. |  |  |  |  |  |  |  |
| I’m open to working with colleagues in new ways to [palliative care best practice]. |  |  |  |  |  |  |  |
| I will continue to support [palliative care best practice]. |  |  |  |  |  |  |  |
| I can easily integrate [palliative care best practice] into my existing work. |  |  |  |  |  |  |  |
| [Palliative care best practice] disrupts working relationships. |  |  |  |  |  |  |  |
| I have confidence in other people’s ability to [palliative care best practice]. |  |  |  |  |  |  |  |
| Work is assigned to those with skills appropriate to [palliative care best practice]. |  |  |  |  |  |  |  |
| Sufficient training is provided to enable staff to [palliative care best practice]. |  |  |  |  |  |  |  |
| Sufficient resources are available to support [palliative care best practice]. |  |  |  |  |  |  |  |
| Management adequately supports [palliative care best practice]. |  |  |  |  |  |  |  |
| I am aware of reports about the effects of [palliative care best practice]. |  |  |  |  |  |  |  |
| The staff agree that [palliative care best practice] is worthwhile. |  |  |  |  |  |  |  |
| I value the effects that [palliative care best practice] has had on my work. |  |  |  |  |  |  |  |
| Feedback about [palliative care best practice] can be used to improve it in the future. |  |  |  |  |  |  |  |
| I can modify how I work with [palliative care best practice]. |  |  |  |  |  |  |  |

Table: Mean scores (SD) by Normalization Process Theory construct, subconstruct and palliative care best practice

| **Construct** | **Subconstruct** | **Identifying Seriously Ill** | | **Advance Care Planning** | | **Palliative dialysis** | |
| --- | --- | --- | --- | --- | --- | --- | --- |
|  |  | **Early^1^** | **Late^2^** | **Early** | **Late** | **Early** | **Late** |
| Coherence | Coherence average  Differentiation  Communal Specification  Individual specification  Internalization | 4.1(0.8)  3.9(0.8)  3.8(1)  4(0.8)  4.5(0.6) | 4.3(1)  4.1(1)  4.1(1)  4.3(1)  4.6(0.9) | 4.1(0.8)  4.1(0.9)  3.7(0.8)  4.3(0.7)  4.4(0.5) | 4.2(0.8)  4.2(0.8)  3.8(0.9  4.5(0.6)  4.4(0.6) | 3.9(0.9)  4.2(0.6)  3.4(1.1)  3.8(0.8)  4(0.6) | 4.1(0.8)  4.5(0.6)  3.5(0.9)  4.1(0.6)  4.3(0.7) |
| Cognitive Participation | Cognitive Participation average  Initiation  Legitimation  Enrollment  Activation | 4.4(0.7)  4(1)  4.4(0.6)  4.6(0.6)  4.6(0.5) | 4.5(0.9)  4.4(0.8)  4.6(0.8)  4.4(1)  4.5(0.9) | 4.3(0.6)  4.1(0.8)  4.3(0.5)  4.4(0.6)  4.5(0.6) | 4.5(0.7)  4.3(0.9)  4.5(0.6)  4.6(0.5)  4.7(0.5) | 4(0.7)  3.7(0.7)  3.9(0.6)  4.3(0.6)  4.3(0.6) | 4.4(0.8)  4.2(1)  4.4(0.7)  4.5(0.6)  4.4(1) |
| Collective Action | Collective Action average  Interactional workability  Relational Integration  Skill set workability  Contextual integration | 4(0.9)  4.3(0.6)  4.2(0.8)  3.6(1)  3.9(0.9) | 4(1.1)  4.3(0.9)  4.1(1)  3.6(1.1)  3.9(1.2) | 3.8(0.9)  4.1(0.7)  4(0.7)  3.6(1)  3.6(1) | 3.9(1)  4.2(0.8)  4.1(0.9)  3.7(1.1)  3.8(1.2) | 3.4(1)  3.8(0.8)  3.6(1)  3.1(1.2)  3.1(1) | 3.5(0.9)  4(0.8)  3.6(1.1)  3.4(0.9)  3.2(0.9) |
| Reflexive Monitoring | Reflexive monitoring average  Systemization  Communal appraisal  Individual appraisal  Reconfiguration | 4.2(0.8)  3.9(0.9)  4(0.8)  4.3(0.7)  4.4(0.6) | 4.1(0.9)  4.1(1)  3.7(1)  4.3(0.9)  4.2(0.8) | 4(0.7)  3.8(0.7)  4.1(0.7)  4.3(0.7)  4(0.6) | 4.3(0.7)  4.1(0.9)  4.1(0.8)  4.5(0.6)  4.5(0.6) | 3.8(0.8)  3.6(0.8)  3.5(0.8)  3.9(0.8)  4(0.5) | 3.9(0.7)  3.7(0.8)  3.6(0.6)  4.1(0.6)  4.2(0.7) |

^1^Early Implementation period immediately following Learning Session 1

^2^Late implementation period following the Action Period after Learning Session 3
